# A tribute to Paul Kleihues, M.D. (1936–2022)

**DOI:** 10.1007/s00401-022-02446-z

**Published:** 2022-06-13

**Authors:** Otmar D. Wiestler

**Affiliations:** Helmholtz Association of German Research Centers, D-10178 Berlin, Germany

Paul Kleihues, a tycoon and international leader in neuropathology and neurooncology as well as a close friend passed away on March 17th 2022 in Zurich at the age of 85 years. A large family of colleagues from all over the world have closely cooperated with Paul and tremendously benefited from his enormous knowledge, his ideas, his charming personality and dynamic character. As a representative of this group, I would like to pay tribute to Paul’s unique achievements.

Personally, I met Paul during my medical school training in Freiburg im Breisgau back in the late 1970s. As medical students, we had to pass exams in pathology. Due to my interest in brain and brain diseases, I signed up for the neuropathology course and met the charming young professor Paul Kleihues who orchestrated the brain cutting session in a truly inspiring manner. After the successful conclusion of this exercise, Paul asked: “Did you already consider a research project for your medical thesis?” This was the beginning of an extremely fruitful scientific relationship and lifelong friendship.

My research project was a bit unusual since we did not study brain tumors at that time. The topic was “Mechanisms of organ-specific tumor induction in the gastro-intestinal tract by methylating agents”. It involved experimental animals, methylating carcinogens and the DNA from several target organs, which we analyzed for the amount of pro-mutagenic O6-methylguanin. What struck me as exciting from the very first day, however, was the incredible spirit and atmosphere in this young division. Everything focused on fascinating research, no scientific challenge was big enough. Neuropathological diagnostic work played a minor role. Quite unique was Paul’s remarkable gift to identify, recruit and motivate creative young talents, which flourished in his environment.

Andreas von Deimling and Adriano Aguzzi have been among the ambitious medical students whom I met in Freiburg. At the end of our medical school training, we asked the obvious question: does it really make sense to enter such an exotic discipline as neuropathology? Paul gave the answer in the end: his division was simply too attractive and he was a real magnet as well as an inspiring leader. He told us from the very beginning to practice neuropathology would only make sense based on a strong research pillar and taught us to ask ambitious scientific questions driven by challenging research goals and curiosity. Every project can be successfully mastered as long as you try hard enough and apply cutting-edge technology.

Another message Paul conveyed to us: it is important to keep eyes and ears open, to constantly look out for interesting new options and to be mobile. He practiced this himself. One day, Prof. Rolf Zinkernagel from the University of Zurich came to the lab. This was a clear signal that the period in Freiburg would come to an end and that a new challenge would wait for Paul in Switzerland. With incredible energy and passion, he moved and turned the Zürich neuropathology division into a world leading laboratory within a short period of time. In the meantime, my family and I had joined the University of California in San Diego to spend three productive years as a postdoctoral scientist in the United States. Paul maintained a close contact and visited us at San Diego to let us know how exciting the prospects for a new career step would be in Zurich. This is why we returned to his unit in Zurich in 1987. It was very obvious that Paul had established himself extremely well. The faculty elected him as Dean of the Medical School.

On the other hand, it became also clear, that his research on chemical carcinogenesis was not state-of-the-art anymore. Molecular biology approaches opened new research perspectives and cancer research was soon dominated by the analysis of oncogenes and tumor suppressor genes. Many colleagues in this situation would have accepted that they could no longer play at the forefront of their respective field. Paul responded with a remarkable decision. Overnight he chose to move to New York University and spend seven months as a sabbatical professor to learn molecular biology himself and apply it to his own research. For us, this was a unique opportunity to coordinate and chair one of the leading neuropathology institutions at a rather young age.

Following his sabbatical leave, he proudly presented sequencing gels, which he had produced himself. His research program changed dramatically with a new focus on the molecular genetics and molecular neuropathology of human brain tumors. Within a short period, the Zurich lab turned into one of the leading sites for molecular brain tumor research. An amazing number of pioneering studies have been initiated during this time, including the seminal work on the molecular definition of glioblastoma subtypes with highly distinct clinical and biological profiles. Among the many colleagues who made significant contributions, were Andreas von Deimling, Karl Plate and Hiroko Ohgaki.

Another groundbreaking idea, which Paul developed during the New York sabbatical, was his plan to initiate and publish a new neuropathology journal, Brain Pathology. With his unprecedented drive and passion, he managed to start a new type of journal with a highly attractive design, trendy black cover, and a clear focus on cutting-edge review articles on some of the most exciting developments in clinical and molecular brain tumor research and neuropathology. This smart approach conquered a remarkable niche in the neuroscience publication arena. Production and marketing followed rather unconventional routes. His wife Inge was in charge of the distribution business. The journals were prepared for shipping in legendary packaging parties at the Institute for Neuropathology in Zurich. Paul himself was visiting neuropathology and neuroscience conferences as a promoter and sold subscriptions during these events. Brain Pathology has experienced an amazing history and continues to be among the leading journals in both neuropathology and the neurosciences.

During this era, we also recognized a remarkable renaissance in his interest for the diagnosis of CNS tumors. The Brain Tumor Reference Center, which he had established in Freiburg, experienced a revival. Paul now followed in the footsteps of his beloved teacher Klaus Joachim Zülch who had established a WHO classification of brain tumors in the early 1960s in Cologne. Stimulated by this historical achievement, it was decided to invite neuropathology experts from all over the world to brain tumor classification meetings in Zürich in the early 1990s. It was the starting point for an ambitious long-term project with the mission to completely revise the WHO classification of brain tumors and constitutes yet another example for an ambitious endeavor, which achieved fundamental success. The WHO classification, which was later produced under Paul’s leadership at the IARC in Lyon, has turned into a standard volume, which you can find next to the microscope of virtually any surgical neuropathologist. Some of us vividly remember legendary WHO consensus meetings in Zurich, in Lyon and later in Heidelberg. Here, Paul brought his many talents to perfection: as a designer and editor, a diplomatic but self-confident moderator of complex discussions and a smart coordinator of international networks. We could tell wonderful anecdotes from these seminal meetings.

Well in line with his belief that one should always keep eyes and ears open and be open for a new challenge in life his Zurich episode ended in 1993. The WHO became aware of Paul and appointed him as the new Director for the International Agency for Research on Cancer (IARC) in Lyon. IARC was an international institute for cancer epidemiology well known to a small group of epidemiology experts. Over the years, it had lost quite a bit of momentum. We were thrilled to observe how Paul managed to achieve a complete turnaround of this institution within only few years. He changed IARC into a highly vibrant, attractive, internationally recognized research center under the auspices of the WHO. This was particularly remarkable considering that at the beginning of his term in Lyon, he didn’t speak a single word of French.

Paul’s 10 years at the IARC turned out to be another very fruitful and highly productive episode during his unique career. He managed to establish a new network with internationally renowned experts in cancer epidemiology and cancer related public health issues. Based on the exciting experience with several editions of the WHO classification of brain tumors, he decided to publish an entire series of WHO classifications of tumors in a total of twelve volumes. Networks of experts in surgical pathology from all over the world came to Lyon and reached a consensus for the WHO classification in their respective area of surgical pathology. Again, these volumes quickly turned into diagnostic standards, which are widely used all over the world. At the end of his term in Lyon, Paul published the World Cancer Report 2003 in a joint action with Bernie Stewart. This book quickly turned into a classical information source for everybody who wants to get a brief overview about important new developments in cancer research and oncology on a global level. Among the many colleagues who flourished under Paul’s guidance in Lyon, Hiroko Ohgaki played a particularly prominent role. Without her contributions, many of the studies in the molecular genetics of brain tumors and the WHO blue books would not have been possible.

Paul retired from IARC at the end of 2003. This completed a remarkably active academic journey starting as an ambitious young scientist at the Max-Planck-Institute in Cologne all the way down to the final mandate as director of the WHO IARC agency in France. The retirement from IARC was by no means the end of Paul’s scientific career. Soon, he was approached by the Medical Center of Freiburg University and decided to return to Freiburg in 2005. Here, he served as founding director of the new Comprehensive Cancer Center, which needed a visionary and experienced leader. Once more he succeeded in laying the ground for a well-structured, strategically positioned and innovative new center. Many organizations appointed him to prestigious committees and review boards. I vividly remember lively meetings in an international panel of experts, which selected German sites for the new comprehensive cancer center program of the German Cancer Aid (Onkologische Spitzenzentren, Deutsche Krebshilfe) in 2007. This group traveled through Germany for two weeks visiting a number of candidate sites. At these legendary meetings on site, in hotel lobbies and during long train rides Paul developed superb skills in summarizing major conclusions and putting them into a Dictaphone right on the spot.

In 2005 and 2006, he spent a 1-year fellowship period at the distinguished Science College in Berlin where he joined forces with quite a number of fascinating personalities from many different fields of science and culture from all over the world. Here he pursued the ambitious idea to design a digital cancer encyclopedia, with which he was clearly ahead of time. Another fellowship period led him to the National Cancer Institute in Bethesda in the United States. Eventually he returned to the University of Zurich as a senior professor and highly recognized consultant.

Paul maintained a very high level of activity and this was only compromised a few weeks before his death. He was extremely well connected, experienced, inspirational and served as a visionary participant in strategic discussions. His scientific oeuvre is truly outstanding with more than 300 highly cited scientific publications, numerous awards including honorary doctoral degrees from renowned Universities and prestigious leadership functions such as director of the Freiburg University Medical Center, Dean of the Medical School at Zürich University, Director of IARC and as distinguished member of supervisory bodies as well as chair of an amazing number of evaluation and review events.

Among his many talents, the most remarkable has been this incredible gift for identifying, recruiting, and nurturing creative talents. I hardly experienced a colleague with comparable skills in finding talented brains, motivating junior colleagues to join his team and guiding so many team members to remarkable scientific careers. Expectations were always very high and tasks challenging—but everybody could count on Paul in critical situations. It is truly amazing to see how many of his colleagues pursued highly successful professional careers of their own. At times more than half of German neuropathology heads had trained with Paul or members of Paul’s teams. What a phenomenal contribution to science and neuropathology, which will have a lasting impact through generations.

I cannot remember a single boring moment with him. In retrospect, we have often asked: how can an individual master such an amazing number of achievements? What are the secrets of his success? Paul was always searching for new avenues, without any concerns to follow unconventional routes or to change fields and affiliations in regular intervals. As soon as he identified a new goal, it was systematically and rigorously pursued. He had unique skills in building an exciting and stimulating environment with enthusiastic motivation skills. Innovative research always builds on brilliant people. Paul was certainly a particularly unique example for this rare entity. An important message he kept telling us in surgical neuropathology: you can only advance in neuropathology if you give research and science-based approaches clear priority. He has been the shining example for implementing this principle.

**Fig. 1:**
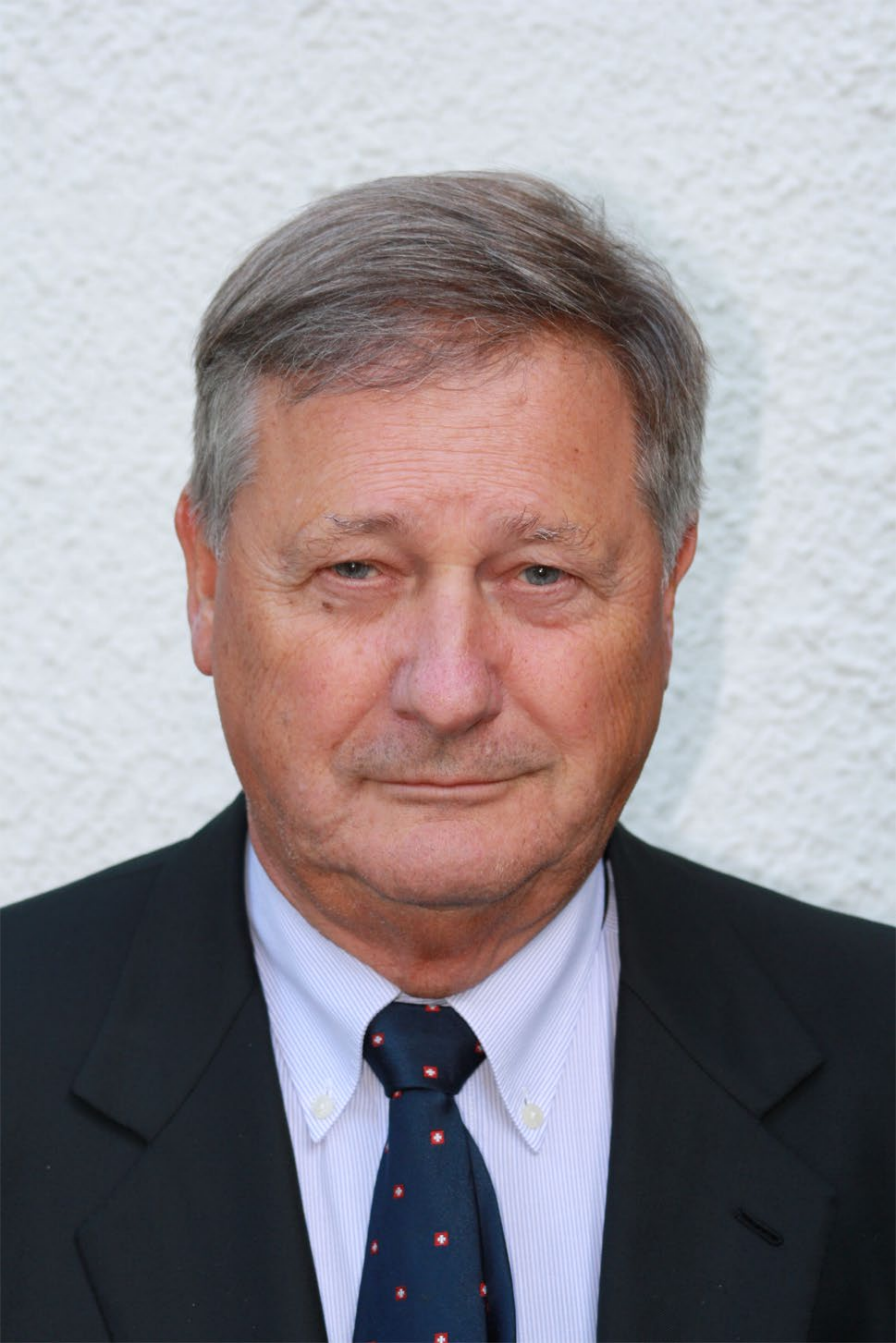
Paul Kleihues

Let us all remember Paul Kleihues as a very special, wonderful man who has shaped the field and who trained an amazing number of international colleagues who will carry his message further into the future. What else can we do for him? Simply keep him alive in our memory and our hearts. In this sense, my dear Paul, you will always be with us (Fig. 1).

